# Measuring needs-based quality of life and self-perceived health inequity in patients with multimorbidity: investigating psychometric measurement properties of the MultiMorbidity Questionnaire (MMQ) using primarily Rasch models

**DOI:** 10.1186/s41687-023-00633-4

**Published:** 2023-09-18

**Authors:** Kristine Bissenbakker, Volkert Siersma, Alexandra Brandt Ryborg Jønsson, Anne Møller, Karl Bang Christensen, John Brandt Brodersen

**Affiliations:** 1 Research Unit for General Practice and Section of General Practice, Department of Public Health, University of Copenhagen, Copenhagen, Denmark; 2 Primary Health Care Research Unit, Region Zealand, Copenhagen, Denmark; 3 Department of People and Technology, Roskilde University, Roskilde, Denmark; 4 Section of Biostatistics, Department of Public Health, University of Copenhagen, Copenhagen, Denmark

**Keywords:** Quality of life, Multimorbidity, Patient-reported outcome measure, Rasch model, Psychometric assessment

## Abstract

**Background:**

Multimorbidity is a burden for the individual and to the healthcare sector worldwide, leading to a rising number of intervention studies towards this patient group. To measure a possible effect of such interventions, an adequate patient-reported outcome measure (PROM) is essential. The aim of this study was to assess the draft MultiMorbidity Questionnaire (MMQ), a PROM measuring needs-based quality of life and self-perceived inequity in patients with multimorbidity, for its psychometric properties and to adjust it accordingly to create a content- and construct valid measure.

**Methods:**

The draft MMQ was sent to 1198 eligible respondents with multimorbidity. Modern test theory and classical test theory were used to analyse data. Dimensionality of the suggested domains and invariance of the items were assessed through item analysis, examining the fit to a psychometric model.

**Results:**

The psychometric analyses were based on responses from 390 patients with multimorbidity. In the MMQ1, measuring needs-based QoL, evidence of six unidimensional scales was confirmed: physical ability (6 items), worries (6 items), limitations in everyday life (10 items), my social life (6 items), self-image (6 items), and personal finances (3 items). The psychometric analyses of the MMQ2 outlined four unidimensional scales measuring the feeling of Self-perceived inequity in patients with multimorbidity: experiences of being stigmatised (4–5 items), Experiences of insufficient understanding of the burden of disease (3 items), Experiences of not being seen and heard (4 items), Experience of powerlessness (5 items). These scales are relevant for patients’ with multimorbidity encounters with (1) their general pratitioner, (2) staff at their general practitioner’s surgery, (3) healthcare professionals, (4) staff at the local authorities and (5) friends, family, and others.

**Conclusion:**

The MMQ, a QoL measure for patients living with multimorbidity has been validated: the MMQ1 is a condition-specific PROM with adequate psychometric properties designed to measure needs-based QoL. The MMQ2 measuring Self-perceived inequity, has also been found to possess adequate measurement properties; however due to the risk of type 2 error a revalidation of MMQ2 is suggested.

**Supplementary Information:**

The online version contains supplementary material available at 10.1186/s41687-023-00633-4.

## Background

The number of adults living with two or more chronic conditions, usually defined as multimorbidity [1], is continuously rising worldwide [2–4]. Besides extended healthcare costs for society [5], qualitative and quantitative studies show how multimorbidity has a negative impact on the individual’s *quality of life* (QoL) [6–8]. To intervene successfully among people living with multimorbidity it is essential to be able to measure the possible effects of studies targeting this patient group. Yet, there is no existing condition-specific patient-reported outcome measure (PROM) for adequately assessing QoL in patients with multimorbidity [9]. Therefore, we have developed the MultiMorbidity Questionnaire (MMQ) for this purpose. In the first stage of developing the MMQ, we found *The needs-based approach to QoL* to be a relevant, but not completely covering, conceptual framework through qualitative interviews with the target group [10–12]. The Needs-based approach to QoL is based on the individual’s possibility of fulfilling their expectations and needs in life [13, 14] and thus, emphasises the importance of involving the target group in generating items [15]. We found it necessary to supplement the conceptual framework with theory on *subjective health inequity*; reactions to how you feel social determinants determine how you are perceived by healthcare professionals [12, 16]. Our studies define this theory as *self-perceived health inequity* [12].

In the second stage of developing the MMQ, we developed items and domains (a set of items measuring nuances of the same construct before psychometric validation) covering the constructs needs-based QoL (MMQ1) and self-perceived inequity (MMQ2) (Tables 1, 2) through qualitative interviews with patients with multimorbidity, ensuring high content validity (content relevance and content coverage) [12].

**Table 1:** Domains and preliminary items of the draft MultiMorbidity Questionnaire (MMQ)

Domains	Number of items
*Needs-based QoL (MMQ1)*	
Physical ability	10
Worries	11
Limitations in everyday life	15
My social life	11
Self-image	12
Personal finances	2
*Self-perceived inequity (MMQ2)*^a^	
Experience of being stigmatised	5
Experience of not being seen and heard	4
Insufficient understanding of the burden of disease	3
Experience of powerlessness	5

^a^The relevant items of the MMQ2 are repeated in encounters with their (1) general practitioner (GP), (2) staff at their GP’ surgery, (3) other healthcare professionals, (4) local authorities, (5) family, friends and others

**Table 2:** Example of an item with response options

My illnesses limit where I can go
□ No, not at all
□ Yes, a little bit
□ Yes, quite a lot
□ Yes, a lot

This article will detail the third stage in the development of the MMQ; psychometric assessment of the developed items and domains using Modern Test Theory (MTT) [17]. The Rasch model, belonging to MTT is regarded as the most strict model when validating PROMs regarding psychometric properties [17–19]. Contrary to classical test theory (CTT), Rasch models test for unidimensionality (i.e. items in a scale measure only a single construct), invariance (i.e. different subgroups do not respond in systematically different ways to a specific item) and local independence (i.e. items within a scale are not correlated beyond the construct measured) [17, 20]. The aim of this study is to test the drafts of the MMQ1 and MMQ2 for their psychometric properties regarding dimensionality of the domains and invariance of the items using MTT and to adjust accordingly. Furthermore, discrimination abilities and reliability of the scales will be tested using CTT.

## Materials and methods

### Stratification of eligible survey respondents

**Fig. 1:**
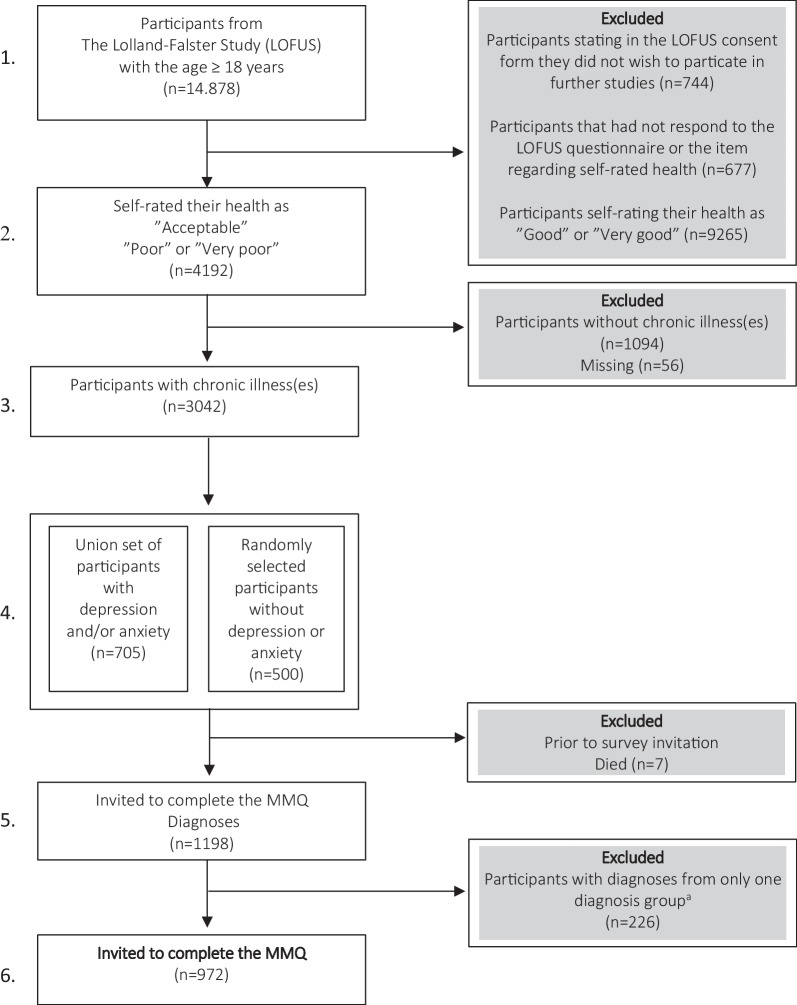
Stratification of eligible survey respondents from the Lolland–Falster health study (LOFUS). Stratification step 1–4 were conducted within the LOFUS database based on the participants' responses to the LOFUS questionnaire. ^a^According to our definition of multimorbidity (see Table 3). ^b^The LOFUS questionnaire: A health related questionnaire completed upon inclusion in the LOFUS [22]

Eligible people invited to respond to the final drafts of the MMQ1 and MMQ2 were adults ≥ 18 years living with multimorbidity. They were recruited via their participation in The Lolland–Falster health study (LOFUS). LOFUS is a household population study of the general population of Lolland and Falster, in two deprived areas of southern Denmark [21]. A stratification among the LOFUS participants was conducted to obtain respondents who we hypothesised to be affected by their chronic conditions on their Needs-based QoL (Fig. 1).

**Table 3:** Diagnoses groups to ensure respondents with multimorbidity (diagnosis from more than one group)

Cardiovascular
Heart thrombus
Arteriosclerosis in the heart
Cardiospasm
Diabetes and sequelae
Diabetes
Kidney disease
Lung
Asthma
Chronic bronchitis, smoker’s lungs (emphysema, chronic obstructive pulmonary disease)
Cancer
Psychical
Anxiety
Depression
Musculoskeletal
Osteoarthritis
Arthritis
Ruptured disc or similar

*Inspired by the definition of multimorbidity by Willadsen et al.* [3]

Excluded participants are listed in Fig. 1 (1–3). Among the remaining LOFUS participants, all participants who had stated to suffer from anxiety and/or depression (N = 698) and a random sample of patients who had stated not to have anxiety or depression (N = 500) were stratified. This step was conducted to ensure enough respondents from the following three groups were invited: (1) Participants with somatic multimorbidity. (2) Participants with psychiatric multimorbidity (anxiety and depression). (3) Participants with psychiatric and somatic multimorbidity. An invitation to complete the MMQ was distributed in December 2019 to the group of 1198 strategically selected LOFUS-participant through a link to SurveyXact via e-Boks (a Danish provider of secure digital post-boxes). A paper version was sent via postal mail to participants without email or e-Boks. A final stratification of eligible respondents was done in the invitation process where participants without multimorbidity were excluded. This was done according to the definition of multimorbidity as suggested by Willadsen et al. “*as having diagnoses from at least 2 of 10 diagnosis groups”* [3] by the respondents’ responses to a list of diagnoses that had also been included in the LOFUS questionnaire. For the purpose of this study, diagnoses were grouped into the diagnosis groups listed in Table 3. Participants who had diagnoses from more than one of the groups and/or both anxiety and depression (N = 972) were invited. Reminders to invited eligible respondents who had not completed the survey within four weeks were distributed in January 2020. In the response to MMQ2 “skip-questions” occurred, enabling the patient to skip questions related to encounters with local authorities and other healthcare professionals if they had no contact to these two groups.

### Psychometric analysis

The data collected were analysed using the Rasch model for polytomous items [23–25]. We evaluated the overall model fit of the draft domains regarding unidimensionality using Andersen’s conditional likelihood ratio test (CLR-χ^2^) [26]. Fit to the Rasch model was assessed for each item using item fit statistics with a known asymptotic distribution [27] and categorised items according to statistical significance (after adjustment for multiple testing). Individual item fit was tested by comparing observed and expected correlations between the individual item score with the rest-score of the remaining items in the scale [28]. Invariance was assessed by Differential Item Function (DIF), testing whether items measured differently among subgroups [19] related to sex (male, female), age groups (< 55, 55–65, > 65) and diagnosis groups (Table 3). This was done using conditional likelihood ratio tests (LRT) comparing nested models. Considering the heterogeneous group of patients with multimorbidity, we removed items with evidence of DIF. Lastly, we tested the items of each domain for local response dependence (LRD), analysing if items were correlated beyond the construct measured in terms of content, structure or wordings [29]. This was done using conditional likelihood ratio tests [30] and where LRD was found, this was accounted for by extending the model loglinear interaction parameters yielding graphical Rasch models [31–34] incorporating LRD. In doing so we compared nested models using conditional likelihood ratio tests (LRT). If items possessed misfit or poor psychometric properties, we tested alternative configurations in a stepwise manner, evaluating which items to remove. These evaluations were based on empirical knowledge from the qualitative interviews [11, 12], clinical experience or theoretical considerations linked to our conceptual frameworks of Needs-based QoL [11, 13, 14] and Self-perceived inequity [12, 16] above results of our analyses. Therefore, some items were kept despite misfit if they possessed high content validity in the qualitative phase [12]. Reliability for each of the resulting scales was assessed using Cronbach’s alpha [35]. To account for multiple testing, we used the Benjamini–Hochberg procedure to control the false discovery rate at 5% [36]. Person fit was evaluated using the conditional probability of response vectors given the observed total scale score and reported as the proportion of respondents with significant misfit and corresponding 95% confidence interval. Targeting was evaluated graphically using person-item location maps.

Discriminative abilities of the MMQ1 scales were determined by comparing the mean (plus SD) sumscores of the scales between the categories of the global item [18, 37] (Table 4); the order of the means should follow the categories’ order. Discriminating ability is calculated as the number of individuals needed in a t-test with 5% significance level to find a clinically meaningful difference in the response categories between known-groups with 80% power; here a difference of 1 point in the response categories of the global item, i.e., between *“very poor”* and *“poor”*, *“poor”* and *“acceptable”*, *“acceptable”* and *“good”*, or *“good”* and *“very good”* was taken as a clinically meaningful difference. A low number of individuals indicates a high discriminating ability of the scale.

**Table 4:** Global item with response categories

When I think about my general needs in life, I think my overall quality of life is:
□ Very good
□ Good
□ Acceptable
□ Poor
□ Very poor

All analyses were conducted using DIGRAM v. 3.05.3 [38, 39] and RUMM2030 software [40]. Figures were made in R v. 4.0.0 [41]. A description of the Rasch analysis implemented in DIGRAM is available in Additional file 1.

## Results

**Fig. 2:**
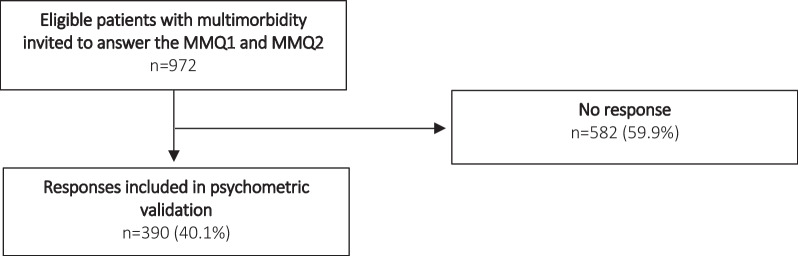
Response rates of the MMQ

From the stratification of eligible respondents in the LOFUS we ensured only patients with diagnoses from different diagnosis groups [3] or more than one psychiatric disease were invited (n = 972). The response rate was 40.1% (Fig. 2). Table 5 shows the characteristics of the surveyed respondents.

**Table 5:** Characteristics of survey respondents

Characteristics/exogenous variable (% of total)	Total number
Total	390 (100)
*Age (years)*	
18–55	119 (30.5)
56–65	120 (30.8)
65+	151 (38.7)
*Gender*	
Female	246 (63.1)
Male	144 (36.9)
*Multimorbidity group*	
Only somatic	117 (30.0)
Only psychiatric	25 (6.4)
Somatic and psychiatric	248 (63.6)
*Diagnoses*^a^	
Heart thrombus	26 (6.7)
Arteriosclerosis in the heart	29 (7.4)
Cardiospasm	35 (9.0)
Diabetes	75 (19.2)
Kidney disease	20 (5.1)
Asthma	66 (16.9)
Chronic bronchitis, smoker’s lungs (emphysema, chronic obstructive pulmonary disease)	69 (17.7)
Anxiety	170 (43.6)
Depression	235 (60.3)
Cancer	69 (17.7)
Osteoarthritis	227 (71.0)
Arthritis	58 (14.9)
Ruptured disc or similar	165 (42.3)
*Education in schoo*l	
7 years or less	54 (13.9)
8–9 years	82 (21.0)
10–11 years	136 (34.9)
Higher general examination (STX)/Higher preparatory examination (HF)	78 (20.0)
Other^b^	37 (9.5)
Missing	3 (0.8)
*Higher education*^c^	
None	61 (15.6)
One or more short courses^d^	34 (8.7)
Vocational education/skilled worker	152 (39.0)
Short-cycle higher education < 3 years	24 (6.2)
Medium-cycle higher education 3–4 years	74 (19.0)
Long-cycle higher education > 4 years	13 (3.3)
Other education	29 (7.4)
Missing	3 (0.8)

^a^Distribution of diagnoses based on the stratification of eligible respondents (see Fig. 2)

^b^Including school abroad

^c^Education after high school or such like

^d^For example specialist working courses, labour courses

### Multimorbidity Questionnaire 1

In the psychometric analysis of the responses to the MMQ1, completed by patients with multimorbidity, we removed a total of 23 items and found that the remaining 37 items had adequate fit to unidimensional Rasch models: 1. Physical ability (6 items), 2. Worries (6 items), 3. Limitations in everyday life (10 items), 4. My social life (6 items), 5. Self-image (6 items), and 6. Personal Finances (3 items). Two items were moved from the domain they originated and tested within another domain. The results from the Rasch analysis for each of these six scales will be described in detail below. The top panel in Table 6 presents fit statistics, Cronbach’s alpha, Person Separation Indices, and proportion with misfit for each of the scales in MMQ1. Table 7 shows the structure and scale profiles of the final MMQ.

**Table 6:** Fit statistics, Cronbach’s alpha, number of respondents and proportion with significant person misfit for each scale in the MMQ1 and MMQ2 after removing items with poor fit or DIF

Scale	No. of items	Item pairs with LRD^a^	Respondents (N)	Fit to graphical Rasch model			Cronbach’s alpha	PSI^*c*^	Proportion with misfit (95% CI)
				CLR-X^2^	*df*	*P*^b^			
*MMQ1*									
1. Physical ability	6	2	345	22.1	30	0.8504	0.86589	0.81172	8.7% (5.7% to 11.7%)
2. Worries	6	3	320	56.8	42	0.0628	0.87617	0.84896	6.9% (4.1% to 9.6%)
3. Limitations in everyday life	10	3	332	90.4	73	0.0818	0.92304	0.88394	8.7% (5.6% to 11.7%)
4. My social life	6	2	295	19.7	35	0.9829	0.87450	0.74587	5.4% (2.8% to 8.0%)
5. Self-image	6	4	301	65.6	52	0.0975	0.89459	0.74587	6.0% (3.3% to 8.9%)
6. Personal finances	3	1	224	10.1	17	0.9013	0.86525	0.59626	3.1% (0.8% to 5.4%)
*MMQ2*									
In encounter with: The general practitioner									
7. Experience of being stigmatised	5	–	126	27.9	14	0.0146	0.88408	–	–
8. Experience of not being seen and heard	4	–	117	29.3	11	0.0020	0.95804	–	–
9. Experience of insufficient understanding of the burden of disease	3	–	125	3.3	8	0.9170	0.97467	–	–
10. Experience of powerlessness	5	–	143	15.3	14	0.3554	0.95682	–	–
Staff at the general practitioner’s surgery									
11. Experience of being stigmatised	5	–	73	5.4	13	0.9653	0.89769	–	–
12. Experience of not being seen and heard	4	–	66	7.8	11	0.7300	0.92431	–	–
13. Experience of insufficient understanding of the burden of disease	3	–	54	1.2	8	0.9963	0.97630	–	–
Other healthcare professionals									
14. Experience of being stigmatised	5	–	47	18.7	13	0.1313	0.83708	–	–
15. Experience of not being seen and heard	4	–	50	8.5	11	0.6696	0.88595	–	–
16. Experience of insufficient understanding of the burden of disease	3	–	50	2.4	8	0.9677	0.97037	–	–
17. Experience of powerlessness	5	–	79	22.3	13	0.0507	0.86294	–	–
Local authorities									
18. Experience of being stigmatised	5	–	53	17.5	14	0.2303	0.91315	–	–
19. Experience of not being seen and heard	4	–	53	19.9	10	0.0307	0.90314	–	–
20. Experience of insufficient understanding of the burden of disease	3	–	51	10.7	8	0.2204	0.98124	–	–
21. Experience of powerlessness	5	–	64	24.4	14	0.0409	0.95179	–	–
Family, friends and others									
22. Experience of being stigmatised	4	–	134	21	11	0.0329	0.85922	–	–
23. Experience of insufficient understanding of the burden of disease	3	–	165	8.4	8	0.3974	0.95969	–	–

^a^Local Response Dependency (LRD). Indicating the number of items within each scale that are correlated beyond the construct measured in terms of content, structure or wordings

^b^The Benjamini–Hochberg adjusted significance levels rejects fit below the usual significance level of 0.05 to account for spurious significant results due to multiple testing. The adjusted *p* value is calculated for each scale fit to the Rasch Model

^c^Person Separation Index (PSI) for each scale. A reliability index that is indicative of the power of the construct to discriminate amongst respondents where 0.7 is minimum. PSI and proportion with misfit have not been reported due to the low sample size of MMQ2

**Table 7:** Structure and scale-score profiles of the MultiMorbidity Questionnaire (MMQ)

Concept	Scale	Number of items	Scale-score profile
*MultiMorbidity Questionnaire 1 (MMQ1)*			
Needs-based quality of life			
	Physical ability	6	0–18
	Worries	6	0–18
	Limitations in everyday life	10	0–30
	My social life	6	0–18
	Self-image	6	0–18
	Personal finances	3	0–9
*MultiMorbidity Questionnaire 2 (MMQ2)*			
Self-perceived inequity^a^			
	Experience of being stigmatised	5	0–15
	Experience of not being seen and heard	4	0–12
	Experience of insufficient understanding of the burden of disease	3	0–9
	Experience of powerlessness	5	0–15

^a^The relevant items of the MMQ2 are repeated in encounters with their (1) general practitioner (GP), (2) staff at their GP’ surgery, (3) other healthcare professionals, (4) local authorities, (5) family, friends and others

In Additional file 2, content condensates of the included and excluded items of the draft MMQ1 and MMQ2 can be found. Additional file 3 shows individual item fits. In the following the stepwise elimination of problematic items are summarised.

#### Physical ability

The draft 10-item domain did not fit the Rasch model. Items 1b (constantly tired), 1c (aware of the body), and 1i (difficulties taking care of body) were removed because of DIF (Table A in Additional file 3) diagnosis. Item 1b, with its content regarding tiredness, was evaluated as too general and therefore could be dispensed. The remaining items indicated no DIF, but item 1a (manages very little) had poor fit (Table A in Additional file 3) and was removed. The remaining six items (1d, 1e, 1f, 1g, 1h, 1j) were found to fit a graphical Rasch model with evidence of LRD for two item pairs (1d and 1j), and (1f and 1g) (Table 6).

#### Worries

The Rasch model rejected the draft 11-item domain. In a stepwise procedure, the following items were removed due to DIF and poor item fit: (1) Item 2k (closest relatives being worried), (2) Item 2d (worried about personal economy), (3) Item 2f (worried about status in society) (Table A in Additional file 3). The wording of Item 2d only focuses on economy and was moved to another domain. Item 2f was also moved to another domain. Two items 2g (feeling mentally vulnerable) and 2h (limited in feeling mentally well) were removed due to DIF (Table A in Additional file 3). The remaining 6 items (2a, 2b, 2c, 2e, 2i, 2j) showed good fit to a graphical Rasch model with LRD for three item pairs (2a and 2c; 2b and 2c; 2e and 2i) (Table 6).

#### Limitations in everyday life

The draft 15-item domain did not fit a graphical Rasch model. Five items were removed stepwise due to DIF and/or misfit: Items 3f (have to plan activities from end to end), 3o (prevented from following what happens in the world), 3j (difficult performing one’s job), 3m (difficult keeping one’s home as one wish to present it to others), and 3g (everyday life planned around illnesses) (Table A in Additional file 3). Two of these (3m and 3g) had content similar to two retained items (3l and 3e). This improved the psychometric properties of the scale and the resulting 10-item scale (3a, 3b, 3c, 3d, 3e, 3h, 3i, 3k, 3l, 3n) fitted a graphical Rasch model with LRD for three item pairs (Table 6). Item 3c showed borderline problematic item fit (*p* = 0.0309) but was retained because of high content validity. Five items showed borderline significant DIF (3a, with respect to age; 3b, with respect to diagnosis; 3e, 3l, 3n, with respect to sex). The *p* values ranged between 0.0181 and 0.0335. These items were also retained due to high content validity.

#### My social life

The draft 11-item domain was rejected by Rasch analysis. The five items 4b (relationship with loved ones affected), 4g (difficulties being anything to others), 4i (difficulties helping loved ones), 4j (limited sex life), and 4k (prevented from sexual activities) were removed due to DIF and/or misfit (Table A in Additional file 3). The two latter showed a high degree of LRD due to their wordings regarding sex as the only items. Omitting these poor-fitting items resulted in a 6-item scale (4a, 4c, 4d, 4e, 4f, 4h) with adequate fit to a graphical Rasch model without DIF, but with LRD between the two item pairs (4d and 4e; 4c and 4h) (Table 6).

#### Self-image

The draft 12-item domain showed poor fit to a graphical Rasch model. The following items were removed due to misfit and/or DIF: 5h (hides illness), 5i (angry at oneself because of illnesses), 5j (disappointed in oneself), 5k (losing one’s role related to employment situation), 5l (losing one’s role in the family) (Table A in Additional file 3). This resulted in a 6-item scale fitted a graphical Rasch model without DFI, but with LRD for four item pairs (5a and 5c; 5d and 5g; 5d and 5e; 5f and 5g) (Table 6).

#### Personal finances

Due to their content relating to personal finances of the omitted items 2d and 2f from the scale “Worries”, these items were added to the suggested 2-item domain “Personal economy”. A single item (6b; prevented from living as one wish to) was removed due to DIF (Table A in Additional file 3). The resulting 3-item scale (6a, 2d, 2f) fitted a Rasch model without DIF, but with LRD between the items 6a and 2f (Table 6).

#### Reliability

The Cronbach’s alpha coefficients for the six MMQ1 scales ranged from 0.87 to 0.92 (Table 6). The Person Separation Index varied from 0.60 to 0.88.

#### Person fit, targeting and discrimination

**Fig. 3:**
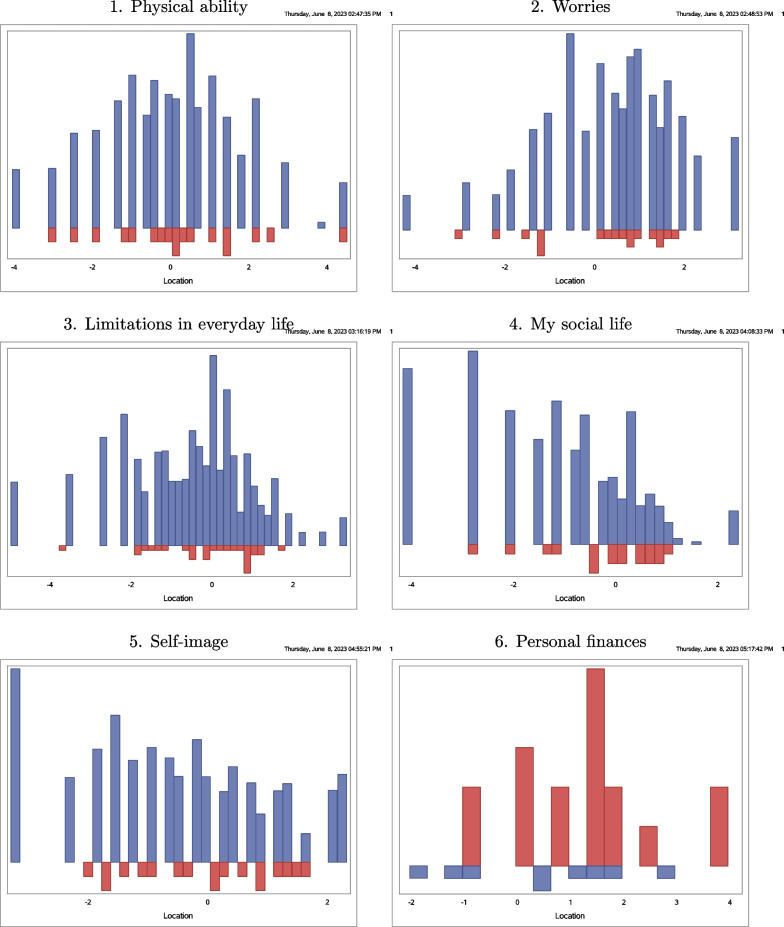
Person-item location maps

**Fig. 4:**
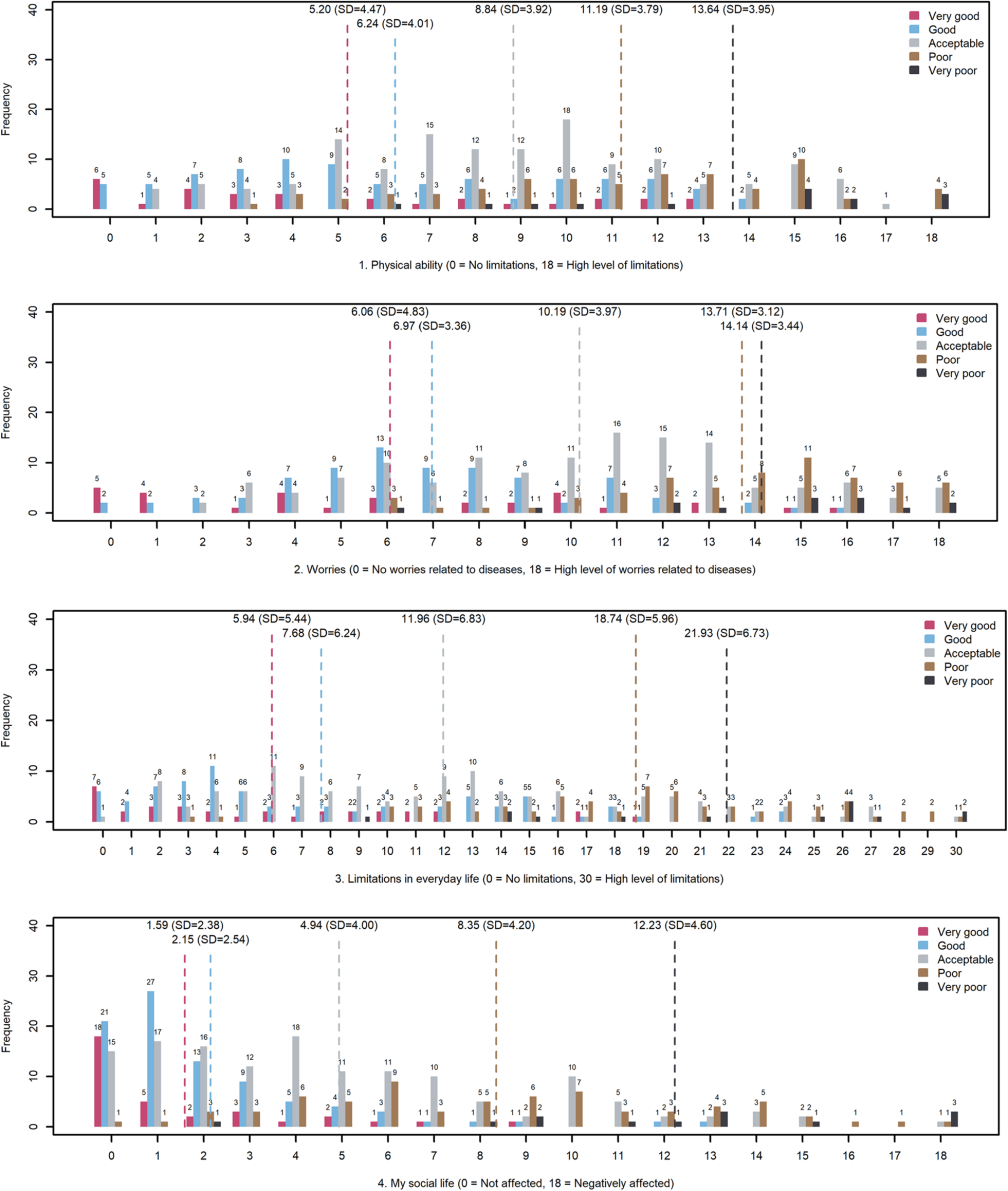

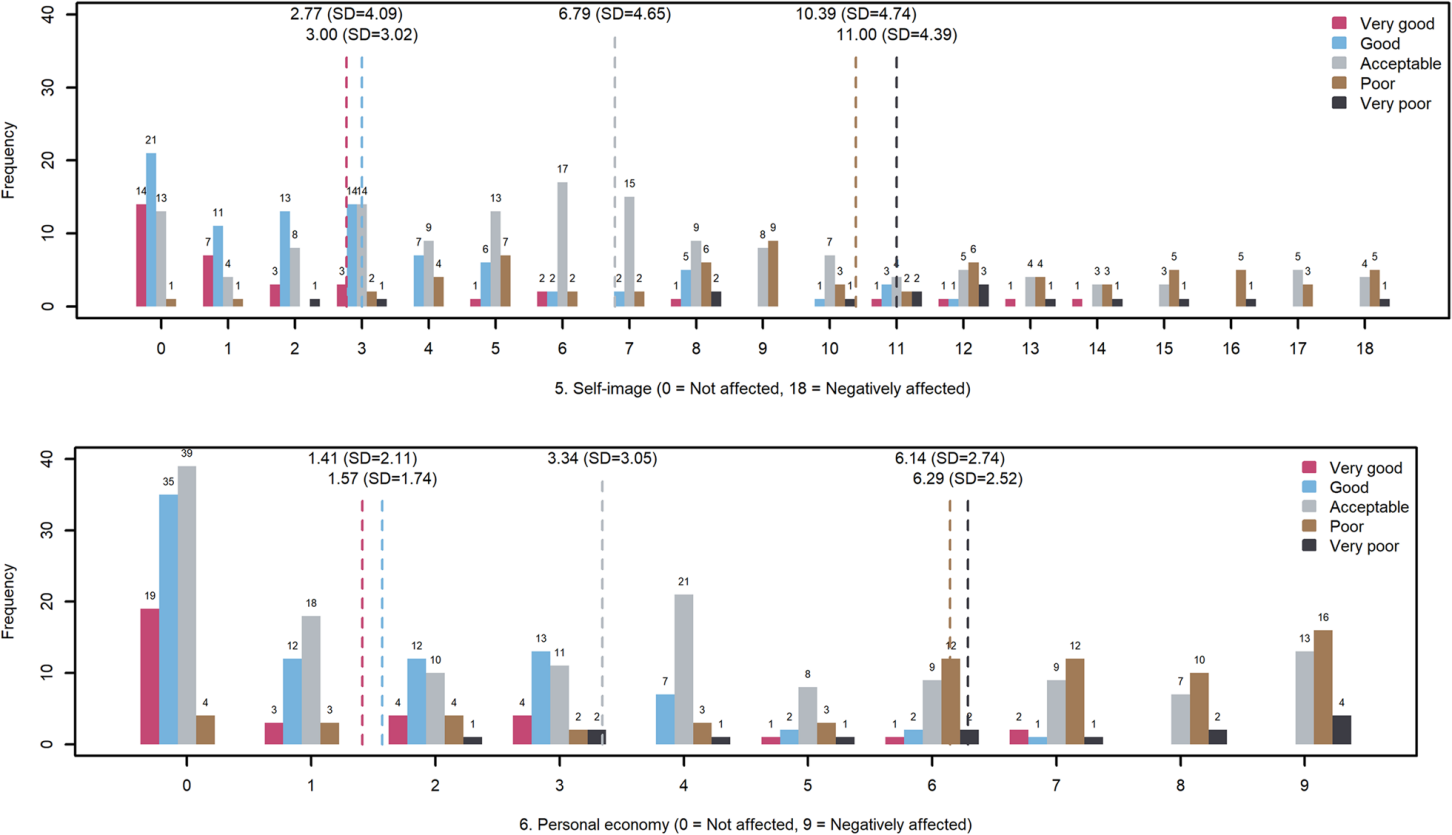
Discrimination abilities of the MMQ1 scales. Responses to each category of the global item within each possible scale-score. X-axis: Total score on the scale. Physical ability as an example: Low score, low perceived limitation). Y-axis: Number of people affirming each category of the global item

The level of person fit was acceptable for all scales and so was the targeting (Fig. 3). The scale 3. Limitations in everyday life performed best in discrimination between the response options *“Acceptable”* and *“Poor”* of the global item. Generally, all the scales performed best in the range between *“Poor”* and *“Good”*. The discrimination abilities for each scale in the MMQ1 corresponding to a difference in the response options of 1 point are presented in Table 8 and illustratively in Fig. 4.

**Table 8:** Discriminative abilities of the MMQ1 scales between the global item’s response options

	Very good–good	Good–acceptable	Acceptable–poor	Poor–very poor
1. Physical ability	582	78	90	84
2. Worries	888	50	42	2012
3. Limitations in everyday life	406	82	34	142
4. Social life	648	68	50	48
5. Self-image	9930	50	58	1898
6. Personal finances	5462	96	40	10,478

### Multimorbidity Questionnaire 2

The psychometric analyses of the MMQ2 outlined the measurement of self-perceived inequity in four unidimensional scales showing fit to the Rasch model, apart from the misfits detailed below. The scales include: 7. Experiences of being stigmatised, 8. Experiences of not being seen and heard, 9. Experiences of insufficient understanding of the burden of disease, and 10. Experience of powerlessness (repeated for encounters with the following groups: (1) The general practitioner (GP), (2) staff at the GP’ surgery, (3) local authorities, (4) other healthcare professionals, (5) friends, families, and others. The lower panel in Table 6 presents fit statistics and Cronbach’s alpha for each of the scales in MMQ1. The following describes the stepwise analyses.

#### In encounters with the GP

##### Experiences of being stigmatised

The draft 5-item scale had acceptable fit to the Rasch model after adjustment for multiple testing (Table 6). Poor item fit was present for items 7c (Pigeonholed because of society’s norms) and 7e (feeling labelled as a second-class citizen) (Table B in Additional file 3). Item 7b (feeling judged because of social status) possessed DIF regarding age (LRT: *p* = 0.0278).

##### Experiences of not being seen and heard

The draft 4-item scale was disregarded by the Rasch model (Table 6). Item 8b (feeling unfairly treated) had poor fit (Table B in Additional file 3), and item 8c presented DIF in terms of age (LRT: *p* = 0.0199).

##### Experiences of insufficient understanding of the burden of disease

The analyses supported the overall fit of the draft 3-item scale to the Rasch model (Table 6). There was evidence of DIF regarding diagnosis for item 9a (the GP having difficulties understanding problems) (LRT*: p* = 0.0006).

##### Experience of powerlessness

The draft 5-item domain was found to fit the Rasch model with no DIF. Item 10c revealed poor item fit (Table B in Additional file 3).

#### In encounters with staff at the GP’s surgery

##### Experiences of being stigmatised

The draft 5-item domain fitted the Rasch model (Table 6). DIF with regards to diagnoses was evident for items 11c (pigeonholed because of society’s norms) (LRT*: p* = 0.0176) and 11e (feeling labelled as a second-class citizen) (LRT: *p* = 0.0156). Furthermore, DIF was present for item 11b (feeling labelled due to a lack of affiliation to the labour market) regarding sex (LRT: *p* = 0.0009).

##### Experiences of not being seen and heard

The draft 4-item scale showed good fit to the Rasch model (Table 6) with no indication of DIF.

##### Experiences of insufficient understanding of the burden of disease

The analyses supported the overall fit of the draft 3-item scale to the Rasch model. The item 13a (staff at the GP’s surgery having difficulties understanding problems) possesed DIF in terms of age group (LRT: *p* = 0.0031) and sex (LRT: *p* = 0.0032).

#### In encounters with other healthcare professionals

There were 165 respondents stating they did not have contact with other healthcare professionals (e.g., hospital department), and therefore did not respond to the items in this domain.

##### Experiences of being stigmatised

The draft 5-item domain possessed fit to the Rasch model with no evidence of DIF (Table 6).

##### Experiences of not being seen and heard

The draft 4-item scale fitted a Rasch model (Table 6). Item 15c (not feeling seen) exhibited DIF regarding age (LRT: *p* = 0.0109).

##### Experiences of insufficient understanding of the burden of disease

The analyses supported the overall fit of the draft 3-item scale to the partial credit Rasch model with no evidence of DIF (Table 6).

##### Experience of powerlessness

The draft 5-item domain was found to fit a Rasch model with no DIF (Table 6).

#### In encounters with local authorities

There were 218 respondents stating they did not have contact with the local authorities (e.g., job centre or case worker), and therefore did not respond to the items in this domain.

##### Experiences of being stigmatised

The draft 5-item domain possessed fit to the Rasch model (Table 6). DIF was present for Item 18e (feeling labelled as a second-class citizen) in terms of age (LRT: *p* = 0.0075).

##### Experiences of not being seen and heard

The analyses supported the overall fit of the draft 4-item scale to the Rasch model (Table 6). Item 19c (not feeling seen) showed DIF regarding sex (LRT: *p* = 0.0163).

##### Experiences of insufficient understanding of the burden of disease

The analyses supported the overall fit of the draft 3-item scale to the partial credit Rasch model with no evidence of DIF (Table 6).

##### Experience of powerlessness

The draft 5-item domain fitted the Rasch model (Table 6). DIF was revealed regarding age group (LRT: *p* = 0.0213). Item 21b (using a lot of energy fighting ‘my cause’) showed poor item fit (Table E in Additional file 3).

#### When spending time with friends, family, and others

##### Experiences of being stigmatised

The draft 4-item scale possessed fit to the Rasch model with no evidence of DIF (Table 6).

##### Experiences of insufficient understanding of the burden of disease

The analyses supported the overall fit of the draft 3-item scale to the partial credit Rasch model with no evidence of DIF (Table 6).

#### Reliability

Reliability was evaluated using Cronbach’s alpha coefficients after evidence of unidimensionality of each scale was established, values ranging from 0.84 to 0.98.

## Discussion

Evidence of six unidimensional scales measuring Needs-based Quality of Life in the MMQ1 were confirmed: Physical ability, Worries, Limitations in everyday life, My social life, Self-image and Personal economy encompassing in total 31 items. In the MMQ2, measuring Self-perceived inequity, four unidimensional scales were outlined: Experiences of being stigmatised (4–5 items), Experiences of not being seen and heard (4 items), Experiences of insufficient understanding of the burden of disease (3 items), and Experience of powerlessness in encounters with (1) the GP, (2) staff at the GP’s surgery, (3) other healthcare professions, (4) local authorities and (5) family, friends, and others (in total a maximum of 16–17 items).

In the psychometric assessment, 23 items were removed from the MMQ1, mainly because of DIF of the individual item regarding diagnoses or age group. This could be expected, considering the responses stemmed from the heterogeneous group of patients with multimorbidity, with different combinations of diagnoses, different severities of illnesses, treatment regimens, and ages. However, we raised evidence that invariant measure was achieved in 6 unidimensional scales encompassing 31 items and thereby comparison of Need-based quality of life across different diagnoses combination in patients living with multimorbidity is possible. Furthermore, as the final MMQ1 only possesses DIF in the scale Limitations in everyday life, it is possible to use this measure in non-randomised studies. In this context, the DIF revealed in this scale requires attention in future studies.

As we have no external objective parameter to compare the MMQ1’s measurement properties we assessed how the individual MMQ1 scales discriminated between the response options of the global item. This differed for each scale and response category, ranging from 34 participants necessary to able to measure the difference between the response options *“Acceptable”* and *“Poor”* in the scale Limitations in everyday life to 10,478 participants in the scale “Personal finances” between the response categories *“Poor”* and *“Very poor”*. Generally, the six MMQ1 scales discriminated best between the response options *“Good”* and *“Poor”*. We interpret this as due to the Danish wordings of the response options in the fare ends of the scales (*“Very poor”* to *“poor”* and *“good”* to *“very good”*) are similar in the context of the items they refer to thereby preventing the ability to discriminate responses. Another interpretation is, that distinguishing in the fare ends of the scales for the individual respondent, is questionable and possibly caused by other day-to-day factors than the latent construct.

Due to a low number of responses to the MMQ2 among the 390 responders, the analyses were based on between 47 and 165 responses for each domain (Table 6), no items from the MMQ2 were disregarded because of insufficient statistical power to detect potential problems, e.g., multidimensionality, misfit, LRD or DIF. Therefore, we suggest a revalidation of MMQ2 among a larger sample size.

Experiences of stigma in encounters with healthcare services are frequently described in vulnerable populations [12, 16]. To the best of our knowledge, no measure exists for measuring these perceptions and the influence on QoL in patients with multimorbidity. In our qualitative item generation phase, this aspect of QoL proved to be of great importance to our informants and resulted in the inclusion of measuring Self-perceived inequity through the construction of four domains, with relevant items repeated for the informants encounters with healthcare professionals, local authorities and family/friends [11, 12]. Rasch analyses indicated that the domains behaved as we expected, yet with the risk of type 2 errors due to low power from the small sample size.

## Strengths and limitations

The primary strength of this study is the content-validity-driven approach of using psychometric methods confirmatory supported by the preliminary conceptual and qualitative phases [11, 12]. This process enables development of scales and items with both high content validity and adequate psychometric properties. Furthermore, the thorough stratified selection of eligible respondents providing the data is a strength for the psychometric assessment. Another strength is the assessment of the discriminating ability of each MMQ1 scale providing evidence of the scales’ measurement capabilities and enabling power calculations for future clinical trials and surveys.

The limitation of this study is primarily related to the low response rate the analyses of the MMQ2 were based on, questioning the applicability directly to other studies. Therefore, to be transparent in the process of psychometrically testing the MMQ2, we thoroughly reported each psychometric flaw the analyses revealed towards unidimensionality, item misfit and DIF. This will guide our revalidation and assessment of the MMQ in a large Danish survey study encompassing more than 1600 responses.

The lower response rates of the MMQ2 compared to the MMQ1 could be related to the response burden. MMQ2 was at the end of the questionnaire, and the same items are repeated for each contact. Yet, the lowest number of responses is due to the possibility of skipping items, if they were not relevant for the respondent, this applied to encounters with local authorities (N = 218) and other healthcare professionals (N = 165). In future studies, the MMQ1 and MMQ2 can be used independently and only encounters relevant for the specific study has to be included.

## Conclusion

This study has raised evidence of adequate psychometric properties, including unidimensional scales encompassing items possessing invariant measurement properties, of the MMQ, a condition-specific PROM for patients living with multimorbidity. The MMQ1, measuring Needs-based QoL, encompasses six scales: Physical ability (6 items), Worries (6 items), Limitations in everyday life (10 items), My social life (6 items) Self-image (6 items), and Personal finances (3 items)—in total 31 items.

Furthermore, the psychometric analyses of the MMQ2 outlines four scales measuring Self-perceived inequity for patients with multimorbidity: experiences of being stigmatised (4–5 items), Experiences of insufficient understanding of the burden of disease (3 items), Experiences of not being seen and heard (4 items), Experience of 1owerlessness (5 items)—in total 16–17 items). These scales are relevant in their encounters with their (1) GP, (2) staff at their GP’s surgery, (3) healthcare professionals, (4) staff at the local authorities and (v) friends, family, and others.

## Implications for research

Our initial systematic review revealed that there is no measure of QoL with adequate psychometric properties specifically for patients with multimorbidity [9]. The MMQ1 is a new multidimensional, condition-specific PROM for measuring Needs-based QoL in patients with multimorbidity with high content validity and adequate measurement properties that can be used in observational- and intervention studies. Further assessment of the MMQ, including revalidation of the MMQ2 for measuring Self-perceived health inequity, is forthcoming. As the MMQ was developed in a Danish setting, work is in progress for translating, adapting, and validating the MMQ into an English context.

### Supplementary Information

**Additional file 1.** Rasch analysis implemented in DIGRAM.

**Additional file 2.** Content condensates of the included and excluded items of the draft MMQ1 and MMQ2.

**Additional file 3.** Individual item fit of items in MMQ1 and MMQ2.

**Additional file 4.** Requirements for using the MultiMorbidity Questionnaire.

## Data Availability

The datasets are available upon reasonable request to the corresponding author.
