# Supplementary material for: Measuring needs-based quality of life and self-perceived health inequity in patients with multimorbidity: investigating psychometric measurement properties of the MultiMorbidity Questionnaire (MMQ) using primarily Rasch models
Source: J Patient Rep Outcomes. 2023 Sep 18;7:94. doi: 10.1186/s41687-023-00633-4 (PMC10506990; doi:10.1186/s41687-023-00633-4)
Supplement: Supplementary file 1 — Additional file 1. Rasch analysis implemented in DIGRAM. [file 41687_2023_633_MOESM1_ESM.docx]

**Additional file 1**

**Supplementary material: Parametrization and inference in the graphical Rasch model**

The graphical Rasch model is an extension of the Rasch model that can incorporate differential item functioning (DIF) and local dependence (LD).

Let X=(X_1_, … ,X_k_) denote the vector of item responses, let Z=(Z_1_, … ,Z_m_) denote a vector of covariates (potential DIF variables), and let θ denote the latent variable that the item measure.

The Rasch model for polytomous items assumes local independence

Prob(X=x|θ) = Prob(X_1_=x_1_|θ) … Prob(X_k_=x_k_|θ) (1)

and that the parametric form is

Prob(X_i_=x_i_|θ,Z) = exp(θx_i_+B(i,x_i_))/K_i_(θ,B) (2)

where B is a matrix of item responses and K is a normalizing that does not depend on x. Note that the fact that left side of equation (2) does not depend on Z. This is due to the assumption of no DIF. Combining (1) and (2) yields

Prob(X=x|θ,Z) = exp(θS+Σ_i_B(i,x_i_))/K (3)

Where S=Σ_i_x_i_ is the sum score and K=Π_i_ K_i_(θ,B).

The graphical Rasch model incorporates LD and DIF by expanding the log-linear structure of the probabilities in equation (3). LD between, say, items X_1_ and X_2_ can be included by fitting the model

Prob(X=x|θ,Z) = exp(θS+Σ_i_B(i,x_i_)+D(x_1_,x_2_))/K (4)

where D is a matrix of interaction parameters. The models (3) and (4) are nested and testing (4) against (3) using conditional likelihood ratio test yields a test of LD. Combining the item pairs with LD into single combination items provides a straightforward way of modelling LD. For two items, say, X_1_ and X_2_ this can be done by replacing them with the a item X_1,2_=X_1_+X_2_.

DIF for, say, item X_1_ with respect to the covariate Z_1_ can be included by fitting the model

Prob(X=x|θ,Z) = exp(θS+Σ_i_Bi(i,x_i_)+L(x_1_,z_1_))/K (5)

where L is a matrix of interaction parameters. The models (3) and (5) are nested and testing (5) against (3) using conditional likelihood ratio test yields a test of DIF. Splitting an item provides a straightforward way of modelling DIF.
