# Supplementary material for: Measuring needs-based quality of life and self-perceived health inequity in patients with multimorbidity: investigating psychometric measurement properties of the MultiMorbidity Questionnaire (MMQ) using primarily Rasch models
Source: J Patient Rep Outcomes. 2023 Sep 18;7:94. doi: 10.1186/s41687-023-00633-4 (PMC10506990; doi:10.1186/s41687-023-00633-4)
Supplement: Supplementary file 2 — Additional file 2. Content condensates of the included and excluded items of the draft MMQ1 and MMQ2. [file 41687_2023_633_MOESM2_ESM.docx]

**Additional file 2**

| **Content condensates of items in the final draft of MMQ1 (Needs-based Quality of Life)**  (ad hoc translation from Danish) | |  |
| --- | --- | --- |
| *1. Physical ability* | |  |
|  | 1a. Overkommer lidt [Manages very little] %  1b. Konstant træt [Constantly tired] %  1c. Opmærksom på kroppen [Aware of the body] %  1d. Ked af at kunne klare lidt [Upset about being able to do so little] +  1e. Ingen fysisk aktivitet for fornøjelsen [No physically activity for pleasure] +  1f. Begrænset i personlig hygiejne [Prevented from maintaining personal hygiene] + |  |
|  | 1g. Begrænset i huslige aktiviteter [Prevented from domestic activities] + |  |
|  | 1h. Presser sig selv mentalt [Push themselves physically] +  1i. Svært at varetage kroppen [Difficulties taking care of body] %  1j. Begrænset i at være aktiv [Prevented from being active] + |  |
| *2. Worries* | |  |
|  | 2a. Bekymringer ift. sygdomme [Worries regarding illnesses] +  2b. Bekymringer ift. behandling [Worries regarding treatment] +  2c. Nervøs for fremtiden [Nervous about the future] +  2d. Bekymret for økonomi [Worried about finances] #  2e. Bekymret for fysisk formåen [Worried about physical ability] +  2f. Bekymret for status i samfundet [Worried about being looked down on] # %  2g. Psykisk sårbar [Psychically vulnerable] %  2h. Forhindret i psykisk velbefindende [Prevented from psychical well-being] %  2i. Presser sig selv mentalt [Push themselves mentally] +  2j. Belaster mine nærmeste [Burden to my loved ones] +  2k. De nærmeste bekymret [Loved ones worried] % |  |
| *3. Limitations in everyday life* | |  |
|  | 3a. Begrænset mobilitet [Limited mobility] + |  |
|  | 3b. Begrænset i spontane aktiviteter [Prevented from spontaneous activities] + |  |
|  | 3c. Spærret inde i eget hjem [Trapped at home] + |  |
|  | 3d. Sygdomme påvirker, hvad der er muligt [Illnesses affect what is possible] +  3e. Nødt til at planlægge i god tid [Have to plan well in advance] +  3f. Nødt til at planlægge aktiviteter fra start til slut [Have to plan activities from end to end] %  3g. Hverdag planlagt ud fra sygdomme [Everyday life planned around illnesses] %  3h. Svært at planlægge ud i fremtiden [Difficult planning far in advance] +  3i. Afhængig af andre [Dependent on others] +  3j. Besvær med at udføre ens arbejde [Difficult performing one’s job] %  3k. Mangler nødvendig hjælp [Lack of necessary support] +  3l. Svært at holde ens hjem [Difficult keeping one’s home] +  3m. Svært at holde ens hjem, som man ønsker at præsentere det for andre [Difficult keeping one’s home as one wish to present it to others] %  3n. Svært at dyrke hobbyer [Difficult to pursue hobbies] +  3o. Begrænset overskud til at følge nyheder [Prevented from following what happens in the world] % |  |
| *4. My social life* | |  |
|  | 4a. Begrænset i samvær med andre [Difficult to spend time with others] +  4b. Forhold til de nærmeste belastet [Relationship with loved ones affected] % |  |
|  | 4c. Belastning for andre [Burden to others] +  4d. Mangler hjælp til at håndtere sygdomme [Lack support in managing illnesses] +  4e. Mangler pårørende til hjælp [Lack a close relative for support] +  4f. Begrænset i at få nye bekendtskaber [Prevented in establishing new relationships] +  4g. Svært ved at være noget for andre [Difficulties being anything to others] %  4h. Svært ved at støtte de nærmeste mentalt [Difficulties in being emotional support to my loved ones]+  4i. Svært ved at hjælpe de nærmeste [Difficulties helping loved ones] %  4j. Begrænset sexliv [Limited sex life] %  4k. Begrænset i seksuelle aktiviteter [Prevented from sexual activities] % |  |
| *5. Self-image* | |  |
|  | 5a. Pinligt berørt over begrænsninger [Embarrassed by limitations] +  5b. Sygdomme påvirker selvtilliden [Illnesses affect the confidence] % |  |
|  | 5c. Sygdomme nedsætter selvværd [Illnesses lower self-esteem] +  5d. Ubehageligt at blive set som syg [Unpleasant to be viewed as ill] +  5e. Føler sig sat i bås [Feel judged] + |  |
|  | 5f. Bebrejder sig selv [Blame oneself] +  5g. Dårlig samvittighed over livsstil [Feel guilty about lifestyle] +  5h. Skjuler sygdomme [Hides illnesses] %  5i. Vred på sig selv pga. sygdomme [Angry at oneself because of illnesses] %  5j. Skuffet over en selv [Disappointed in oneself] %  5k. Mister sin rolle på arbejdsmarkedet [Losing one’s role related to employment situation] %  5l. Mister sin rolle i familien [Losing one’s role in the family] % |  |
| *6. Personal finances* | |  |
|  | 6a. Begrænset i mulighed for god økonomi [Prevented from the possibility of having a good economy] +  6b. Begrænset i at leve som man har lyst til [Prevented from living as one wish to] % |  |

+ Item endorsed in the final MMQ1

# Item moved to other domain

% Item removed after psychometric analysis

| **Content condensates of items in the final draft of MMQ2 (Self-perceived health inequity)**  *How patients with multimorbidity perceive being met by their surroundings, items are repeated for the relevant encounters with (ii) their GP, (ii) staff at their GP’s surgery, (iii) healthcare professionals, (iv) staff at the local authorities and (v) family, friends, and others* | |
| --- | --- |
| *Experiences of being stigmatized* | |
|  | Uretfærdigt stemplet pga. sygdomme [Unfairly labelled because of illnesses] |
|  | Uretfærdigt stemplet pga. jobsituation [Unfairly labelled because of employment situation] |
|  | Sat i bås pga. samfundets normer [Pigeonholed because of society’s norms]  Mistillid omkring at følge råd og vejledning [Doubts one’s ability to follow advice and instructions]  *(not repeated for (iv)*  Sat i bås som andenrangsborger [Labelled as a second-class citizen] |
| *Experiences of not being seen and heard (not repeated for (v))* | |
|  | Dårligere behandlet [Treated worse] |
|  | Uretfærdigt behandlet [Unfairly treated]  ”Ikke set” [“Not seen”]  ”Ikke hørt” [”Not heard”] |
| *Experiences of insufficient understanding of the burden of disease* | |
|  | Sundhedspersonale har svært ved at forstå problemer og udfordringer [Healthcare professionals don’t fully understand difficulties]  Sundhedspersonale har svært ved at tage problemer og udfordringer alvorligt [Healthcare professionals don’t take difficulties seriously]  Sundhedspersonale har svært ved at anerkende problemer og udfordringer [Healthcare professionals have a hard time acknowledging difficulties] |
| *Experiences of powerlessness (not repeated for (v))* | |
|  | Ubetydelig brik [Insignificant piece of a jigsaw] |
|  | Kæmper sin sag [Pleads ones case]  Magtesløshed [Powerlessness]  Nedværdiget [Looked down on]  Ydmyget [Humiliated] |
