# Supplementary material for: Measuring needs-based quality of life and self-perceived health inequity in patients with multimorbidity: investigating psychometric measurement properties of the MultiMorbidity Questionnaire (MMQ) using primarily Rasch models
Source: J Patient Rep Outcomes. 2023 Sep 18;7:94. doi: 10.1186/s41687-023-00633-4 (PMC10506990; doi:10.1186/s41687-023-00633-4)
Supplement: Supplementary file 3 — Additional file 3. Individual item fit of items in MMQ1 and MMQ2. [file 41687_2023_633_MOESM3_ESM.docx]

**Additional file 3**

**Individual item fit**

**Table A.** Individual item fit for deleted and retained items of MMQ1

|  |  | **Deleted items** | | | | | | **Retained items** | | |
| --- | --- | --- | --- | --- | --- | --- | --- | --- | --- | --- |
| **Scale** | **Item number and content condensates**  (Ad hoc translations from Danish) | **Fit to Rasch Model** | | | **DIF (P-value)** | | | **Fit to Rasch Model** | | |
|  |  | Observed | Expected | P | age group | diag | sex | Observed | Expected | P |
| 1. Physical ability | 1a. Manages very little | 0.45 | 0.62 | <0.0001 | 0.3225 | 0.5485 | 0.6781 |  |  |  |
|  | 1b. Constantly tired | 0.56 | 0.62 | 0.0859 | 0.0293 | 0.1607 | 0.0143 |  |  |  |
|  | 1c. Aware of the body | 0.61 | 0.62 | 0.6961 | 0.0002 | 0.0190 | 0.0021 |  |  |  |
|  | 1d. Upset about being able to do so little |  |  |  |  |  |  | 0.75 | 0.73 | 0.4347 |
|  | 1e. No physical activity for pleasure |  |  |  |  |  |  | 0.66 | 0.67 | 0.7117 |
|  | 1f. Prevented from maintaining personal hygiene |  |  |  |  |  |  | 0.71 | 0.76 | 0.1360 |
|  | 1g. Prevented from domestic activities |  |  |  |  |  |  | 0.70 | 0.69 | 0.8412 |
|  | 1h. Push themselves physically |  |  |  |  |  |  | 0.72 | 0.66 | 0.0730 |
|  | 1i. Difficulties taking care of body | 0.56 | 0.64 | 0.0301 | 0.0629 | 0.0015 | 0.1912 |  |  |  |
|  | 1j. Prevented from being active |  |  |  |  |  |  | 0.72 | 0.72 | 0.9957 |
| 2. Worries | 2a. Worries regarding illnesses |  |  |  |  |  |  | 0.73 | 0.70 | 0.3617 |
|  | 2b. Worries regarding treatment |  |  |  |  |  |  | 0.67 | 0.69 | 0.4506 |
|  | 2c. Nervous about the future |  |  |  |  |  |  | 0.79 | 0.76 | 0.2979 |
|  | 2d. Worried about finances  (moved to scale 6) | 0.58 | 0.64 | 0.0621 | 0.0000 | 0.0097 | 0.3776 |  |  |  |
|  | 2e. Worried about physical ability |  |  |  |  |  |  | 0.68 | 0.66 | 0.6466 |
|  | 2f. Worried about being looked down on  (moved to scale 6) | 0.58 | 0.65 | 0.0542 | 0.0000 | 0.1202 | 0.6822 |  |  |  |
|  | 2g. Psychically vulnerable | 0.75 | 0.69 | 0.0472 | 0.0772 | 0.0035 | 0.0643 |  |  |  |
|  | 2h. Prevented from psychical well-being | 0.74 | 0.74 | 0.9691 | 0.4719 | 0.0004 | 0.8512 |  |  |  |
|  | 2i. Push themselves mentally |  |  |  |  |  |  | 0.64 | 0.66 | 0.6264 |
|  | 2j. Burden to my loved ones |  |  |  |  |  |  | 0.61 | 0.61 | 0.9025 |
|  | 2k. Loved ones worried | 0.57 | 0.63 | 0.0757 | 0.0567 | 0.0040 | 0.0269 |  |  |  |
| 3. Limitations in everyday life | 3a. Limited mobility |  |  |  |  |  |  | 0.74 | 0.77 | 0.2357 |
|  | 3b. Prevented from spontaneous activities |  |  |  |  |  |  | 0.76 | 0.79 | 0.1623 |
|  | 3c. Trapped at home |  |  |  |  |  |  | 0.79 | 0.72 | 0.0309 |
|  | 3d. Illnesses affect what is possible |  |  |  |  |  |  | 0.79 | 0.74 | 0.0639 |
|  | 3e. Have to plan well in advance |  |  |  |  |  |  | 0.67 | 0.66 | 0.7107 |
|  | 3f. have to plan activities from end to end | 0.70 | 0.78 | 0.0001 | 0.0013 | 0.1292 | 0.1822 |  |  |  |
|  | 3g. Everyday life planned around illnesses | 0.79 | 0.70 | 0.5474 | 0.0011 | 0.2604 | 0.4316 |  |  |  |
|  | 3h. Difficult planning far in advance |  |  |  |  |  |  | 0.68 | 0.66 | 0.4970 |
|  | 3i. Dependent on others |  |  |  |  |  |  | 0.67 | 0.65 | 0.6995 |
|  | 3j. Difficult performing one’s job | 0.70 | 0.65 | 0.1514 | 0.1896 | 0.0259 | 0.0009 |  |  |  |
|  | 3k. Lack of necessary support |  |  |  |  |  |  | 0.63 | 0.65 | 0.6792 |
|  | 3l. Difficult keeping one’s home |  |  |  |  |  |  | 0.64 | 0.66 | 0.7318 |
|  | 3m. Difficult keeping one’s home as one wish to present it to others | 0.68 | 0.76 | 0.0024 | 0.4795 | 0.0150 | 0.8703 |  |  |  |
|  | 3n. Difficult to pursue hobbies |  |  |  |  |  |  | 0.66 | 0.65 | 0.8058 |
|  | 3o. Prevented from following what happens in the world | 0.60 | 0.60 | 0.9893 | 0.0026 | 0.0006 | 0.0314 |  |  |  |
| 4. My social life | 4a. Difficult to spend time with others |  |  |  |  |  |  | 0.75 | 0.69 | 0.0399 |
|  | 4b. Relationship with loved ones affected | 0.67 | 0.67 | 0.86919 | 0.3441 | 0.1446 | 0.0358 |  |  |  |
|  | 4c. Burden to others |  |  |  |  |  |  | 0.65 | 0.67 | 0.6553 |
|  | 4d. Lack support in managing illnesses |  |  |  |  |  |  | 0.75 | 0.77 | 0.4701 |
|  | 4e. Lack a close relative for support |  |  |  |  |  |  | 0.71 | 0.77 | 0.0816 |
|  | 4f. Prevented in establishing new relationships |  |  |  |  |  |  | 0.72 | 0.70 | 0.6274 |
|  | 4g. Difficulties being anything to others | 0.74 | 0.64 | 0.0130 | 0.4081 | 0.3434 |  |  |  |  |
|  | 4h. Difficulties in being emotional support to my loved ones |  |  |  |  |  |  | 0.67 | 0.66 | 0.6790 |
|  | 4i. Difficulties helping loved ones | 0.58 | 0.50 | 0.0714 | 0.4240 | 0.0387 |  |  |  |  |
|  | 4j. limited sex life | 0.54 | 0.74 | <0.0001 | <0.0001 | 0.0010 |  |  |  |  |
|  | 4k. Prevented from sexual activities | 0.53 | 0.74 | <0.0001 | <0.0001 | 0.0002 |  |  |  |  |
| 5. Self-image | 5a. Embarrassed by limitations |  |  |  |  |  |  | 0.76 | 0.72 | 0.1335 |
|  | 5b. Illnesses affect the confidence | 0.75 | 0.68 | 0.0201 | 0.2176 | 0.0073 | 0.0608 |  |  |  |
|  | 5c. Illnesses lower self-esteem |  |  |  |  |  |  | 0.72 | 0.73 | 0.8761 |
|  | 5d. Unpleasant to be viewed as ill |  |  |  |  |  |  | 0.69 | 0.70 | 0.7102 |
|  | 5e. Feel judged |  |  |  |  |  |  | 0.78 | 0.76 | 0.2875 |
|  | 5f. Blame oneself |  |  |  |  |  |  | 0.68 | 0.73 | 0.1086 |
|  | 5g. Feel guilty about lifestyle |  |  |  |  |  |  | 0.65 | 0.64 | 0.6697 |
|  | 5h. Hides illness | 0.63 | 0.69 | 0.0766 | 0.0078 | 0.3681 | 0.0215 |  |  |  |
|  | 5i. Angry at oneself because of illnesses | 0.82 | 0.74 | 0.0012 | 0.3437 | 0.9032 | 0.8566 |  |  |  |
|  | 5j. Disappointed in oneself | 0.82 | 0.74 | 0.0012 | 0.3437 | 0.9032 | 0.8566 |  |  |  |
|  | 5k. Losing one’s role related to employment situation | 0.52 | 0.72 | <0.0001 | <0.0001 | 0.0015 | 0.2635 |  |  |  |
|  | 5l. Losing one’s role in the family | 0.62 | 0.73 | 0.0008 | 0.1960 | 0.0039 | 0.0023 |  |  |  |
| 6. Personal finances | 6a. Prevented from the possibility of having a good economy |  |  |  |  |  |  | 0.77 | 0.77 | 0.9577 |
|  | 6b. Prevented from living as one wishes to | 0.80 | 0.82 | 0.5095 | 0.0013 | 0.5126 | 0.5616 |  |  |  |
|  | 2d. Worried about personal finances |  |  |  |  |  |  | 0.83 | 0.83 | 0.9858 |
|  | 2f. Worried about being looked down on |  |  |  |  |  |  | 0.76 | 0.76 | 0.8754 |

**Table B**. Individual item fit MMQ2 - in encounters with the general practitioner

| Scale | Item number and content condensates  (Ad hoc translations from Danish) | Fit to Rasch Model | | |
| --- | --- | --- | --- | --- |
|  |  | Observed | Expected | P |
| Experience of being stigmatised | | | | |
|  | 7a. Unfairly labelled because of illnesses | 0.88 | 0.86 | 0.3095 |
|  | 7b. Unfairly labelled because of employment situation | 0.86 | 0.86 | 0.7841 |
|  | 7c. Pigeonholed because of society’s norms | 0.75 | 0.85 | 0.0001 |
|  | 7d. Doubt’s one’s ability to follow advice and instructions | 0.87 | 0.85 | 0.4170 |
|  | 7e. Labelled as a second-class citizen | 0.93 | 0.86 | 0.0127 |
| Experience of not being seen and heard | | | | |
|  | 8a. Treated worse | 0.93 | 0.95 | 0.1204 |
|  | 8b. Unfairly treated | 0.97 | 0.95 | 0.0287 |
|  | 8c. “Not seen” | 0.96 | 0.96 | 0.6913 |
|  | 8e. “Not heard” | 0.95 | 0.95 | 0.7205 |
| Experience of insufficient understanding of the burden of disease | | | | |
|  | 9a. Healthcare professionals don’t fully understand difficulties | 0.96 | 0.97 | 0.1041 |
|  | 9b. Healthcare professionals don’t take difficulties seriously | 0.99 | 0.98 | 0.1618 |
|  | 9c. Healthcare professionals have a hard time acknowledging difficulties | 0.98 | 0.98 | 0.6754 |
| Experience of powerlessness | | | | |
|  | 10a. Insignificant piece of a jigsaw | 0.88 | 0.92 | 0.0653 |
|  | 10b. Pleads one’s case | 0.89 | 0.92 | 0.0810 |
|  | 10c. Powerlessness | 0.96 | 0.92 | 0.0108 |
|  | 10d. Looked down on | 0.96 | 0.93 | 0.0668 |
|  | 10e. Humiliated | 0.93 | 0.93 | 0.8079 |

| Scale | Item number and content condensates  (Ad hoc translations from Danish) | Fit to Rasch Model | | |
| --- | --- | --- | --- | --- |
|  |  | Observed | Expected | P |

| Experience of being stigmatised | | | | |
| --- | --- | --- | --- | --- |
|  | 11a. Unfairly labelled because of illnesses | 0.93 | 0.92 | 0.6246 |
|  | 11b. Unfairly labelled because of employment situation | 0.91 | 0.91 | 0.9785 |
|  | 11c.Pigeonholed because of society’s norms | 0.90 | 0.92 | 0.5464 |
|  | 11d. Doubt’s one’s ability to follow advice and instructions | 0.91 | 0.91 | 0.7301 |
|  | 11e. Labelled as a second-class citizen | 0.93 | 0.92 | 0.7561 |
| Experience of not being seen and heard | | | | |
|  | 12a. Treated worse | 0.93 | 0.95 | 0.2547 |
|  | 12b. Unfairly treated | 0.95 | 0.95 | 0.9144 |
|  | 12c. “Not seen” | 0.96 | 0.96 | 0.5665 |
|  | 12d. “Not heard” | 0.96 | 0.95 | 0.5851 |
| Experience of insufficient understanding of the burden of disease | | | | |
|  | 13a. Healthcare professionals don’t fully understand difficulties | 0.99 | 0.99 | 0.7637 |
|  | 13b. Healthcare professionals don’t take difficulties seriously | 0.99 | 0.99 | 0.8809 |
|  | 13c. Healthcare professionals have a hard time acknowledging difficulties | 0.99 | 0.99 | 0.6543 |

**Table C.** Individual item fit MMQ2 - in encounters with staff at the general practitioner’s surgery

| Scale | Item number and content condensates  (Ad hoc translations from Danish) | Fit to Rasch Model | | |
| --- | --- | --- | --- | --- |
|  |  | Observed | Expected | P |

**Table D.** Individual item fit MMQ2 - in encounters with other healthcare professionals

| Experience of being stigmatised | | | | |
| --- | --- | --- | --- | --- |
|  | 14a. Unfairly labelled because of illnesses | 0.85 | 0.90 | 0.1021 |
|  | 14b. Unfairly labelled because of employment situation | 0.90 | 0.89 | 0.8038 |
|  | 14c. Pigeonholed because of society’s norms | 0.86 | 0.89 | 0.2711 |
|  | 14d. Doubt’s one’s ability to follow advice and instructions | 0.92 | 0.89 | 0.4390 |
|  | 14e. Labelled as a second-class citizen | 0.97 | 0.90 | 0.0894 |
| Experience of not being seen and heard | | | | |
|  | 15a. Treated worse | 0.89 | 0.91 | 0.5432 |
|  | 15b. Unfairly treated | 0.93 | 0.91 | 0.3266 |
|  | 15c. “Not seen” | 0.92 | 0.93 | 0.8635 |
|  | 15d. “Not heard” | 0.92 | 0.91 | 0.8022 |
| Experience of insufficient understanding of the burden of disease | | | | |
|  | 16a. Healthcare professionals don’t fully understand difficulties | 0.98 | 0.99 | 0.1902 |
|  | 16b. Healthcare professionals don’t take difficulties seriously | 0.99 | 0.98 | 0.2617 |
|  | 16c. Healthcare professionals have a hard time acknowledging difficulties | 0.99 | 0.99 | 0.4277 |
| Experience of powerlessness | | | | |
|  | 17a. Insignificant piece of a jigsaw | 0.86 | 0.87 | 0.6449 |
|  | 17b. Pleads one’s case | 0.85 | 0.89 | 0.0995 |
|  | 17c. Powerlessness | 0.92 | 0.88 | 0.1832 |
|  | 17d. Looked down on | 0.88 | 0.87 | 0.7612 |
|  | 17e. Humiliated | 0.91 | 0.86 | 0.2168 |

| Scale | Item number and content condensates  (Ad hoc translations from Danish) | Fit to Rasch Model | | |
| --- | --- | --- | --- | --- |
|  |  | Observed | Expected | P |

**Table E.** Individual item fit MMQ2 - in encounters with local authorities

| Experience of being stigmatised | | | | | |
| --- | --- | --- | --- | --- | --- |
|  | 18a. Unfairly labelled because of illnesses | | 0.82 | 0.86 | 0.3293 |
|  | 18b. Unfairly labelled because of employment situation | | 0.86 | 0.86 | 0.8903 |
|  | 18c. Pigeonholed because of society’s norms | | 0.79 | 0.85 | 0.1923 |
|  | 18d. Doubt’s one’s ability to follow advice and instructions | | 0.88 | 0.85 | 0.4696 |
|  | 18e. Labelled as a second-class citizen | | 0.93 | 0.85 | 0.0752 |
| Experience of not being seen and heard | | | | | |
|  | 19a. Treated worse | | 0.89 | 0.88 | 0.7367 |
|  | 19b. Unfairly treated | | 0.88 | 0.88 | 0.9461 |
|  | 19c. “Not seen” | | 0.90 | 0.90 | 0.9814 |
|  | 19e. “Not heard” | | 0.89 | 0.89 | 0.9616 |
| Experience of insufficient understanding of the burden of disease | | | | | |
|  | 20a. Healthcare professionals don’t fully understand difficulties | | 0.98 | 0.98 | 0.8080 |
|  | 20b. Healthcare professionals don’t take difficulties seriously | | 1.00 | 0.98 | 0.1128 |
|  | 20c. Healthcare professionals have a hard time acknowledging difficulties | | 0.98 | 0.98 | 0.7549 |
| Experience of powerlessness | | | | | |
|  | | 21a. Insignificant piece of a jigsaw | 0.89 | 0.89 | 0.9493 |
|  | | 21b. Pleads one’s case | 0.84 | 0.91 | 0.0109 |
|  | | 21c. Powerlessness | 0.91 | 0.89 | 0.5108 |
|  | | 21d. Looked down on | 0.95 | 0.90 | 0.0559 |
|  | | 21e. Humiliated | 0.93 | 0.90 | 0.2884 |

**Table F.** Individual item fit MMQ2 - in encounters with friends, family, and others

| Scale | Item number and content condensates  (Ad hoc translations from Danish) | Fit to Rasch Model | | |
| --- | --- | --- | --- | --- |
|  |  | Observed | Expected | P |

| Experience of being stigmatised | | | | |
| --- | --- | --- | --- | --- |
|  | 22a. Unfairly labelled because of illnesses | 0.79 | 0.83 | 0.1320 |
|  | 22b. Unfairly labelled because of employment situation | 0.87 | 0.84 | 0.2814 |
|  | 22c. Pigeonholed because of society’s norms | 0.81 | 0.84 | 0.3640 |
|  | 22d. Labelled as a second-class citizen | 0.90 | 0.84 | 0.0413 |
| Experience of insufficient understanding of the burden of disease | | | | |
|  | 23a. Healthcare professionals don’t fully understand difficulties | 0.96 | 0.96 | 0.8361 |
|  | 23b. Healthcare professionals don’t take difficulties seriously | 0.96 | 0.96 | 0.9936 |
|  | 23c. Healthcare professionals have a hard time acknowledging difficulties | 0.98 | 0.96 | 0.2393 |
