# Supplementary material for: Measuring needs-based quality of life and self-perceived health inequity in patients with multimorbidity: investigating psychometric measurement properties of the MultiMorbidity Questionnaire (MMQ) using primarily Rasch models
Source: J Patient Rep Outcomes. 2023 Sep 18;7:94. doi: 10.1186/s41687-023-00633-4 (PMC10506990; doi:10.1186/s41687-023-00633-4)
Supplement: Supplementary file 4 — Additional file 4. Requirements for using the MultiMorbidity Questionnaire. [file 41687_2023_633_MOESM4_ESM.docx]

### **Additional File 4**

### **Requirements for using the MultiMorbidity Questionnaire**

The MMQ will be available for non-profit research free of charge, but with the two following specific requirements, if the MMQ is used in other settings regarding context and cultural differences: 1) Translation and adaption should be done using the two-panel method [1]. 2) The PROM should be validated in the specific setting it is to be used using the Rasch model.

1. Swaine-Verdier A, Doward LC, Hagell P, et al (2004) Adapting quality of life instruments. Value Heal 7:S27–S30. https://doi.org/10.1111/j.1524-4733.2004.7s107.x
